# Supplementary material for: Tailored Synthesis of Heterogenous 2D TMDs and Their Spectroscopic Characterization
Source: Nanomaterials (Basel). 2024 Jan 23;14(3):248. doi: 10.3390/nano14030248 (PMC10856291; doi:10.3390/nano14030248)
Supplement: Supplementary file 1 [file nanomaterials-14-00248-s001.zip › nanomaterials-2820301-supplementary.pdf]

nanomaterials

MDPI

Supplementary Data

Tailored Synthesis of Heterogenous 2D TMDs and Their Spectroscopic Characterization

Jungtae Nam [1], Gil Yong Lee [1], Dong Yun Lee [1], Dongchul Sung [1], Suklyun Hong [1], A-Rang Jang [2] and Keun Soo Kim [1,*]

[1] Department of Physics and Graphene Research Institute, Sejong University, Seoul 05006, Republic of Korea; goodnjt@sejong.ac.kr (J.N.); rlfdyd2000@naver.com (G.L.); geovi012@gmail.com (D.L.); dongchul75@sejong.ac.kr (D.S.); hong@sejong.ac.kr (S.H.)

[2] Division of Electrical, Electronic and Control Engineering, Kongju National University, Cheonan 31080, Republic of Korea; arjang@kongju.ac.kr

* Correspondence: kskim2676@sejong.ac.kr; Tel.: +82-2-3408-3988

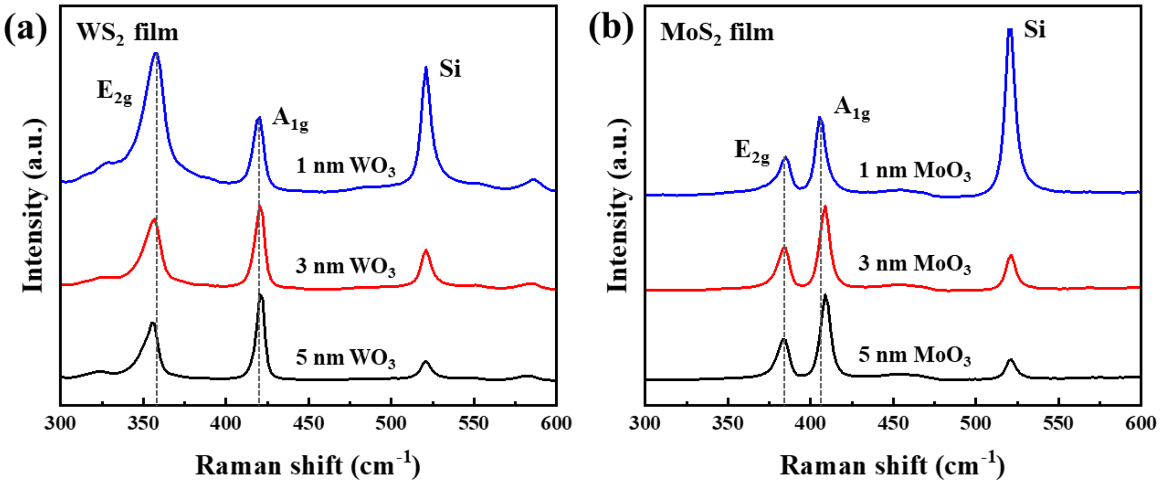

Figure S1. Changes in **(a)** WS_2 and **(b)** MoS_2 Raman spectra according to metal oxide thickness of 1, 3, and 5 nm.

Table S1. Summary of the phonon mode in figure R1 depend on the thickness of the TMO.

TMD	TMO thickness Material	TMO thickness (nm)	Phonon mode (λ = 514 nm) E_{2g} (cm^{-1})	A_{1g} (cm^{-1})	Gap (A_{1g}-E_{2g}) (cm^{-1})	avg. # of layer
WS_2	WO_3	1	358.6	419.5	61.0	~ 2
	WO_3	3	357.2	420.4	63.2	~ 4
	WO_3	5	356.3	420.9	64.6	# > 5
MoS_2	MoO_3	1	384.7	406.0	21.3	~ 2
	MoO_3	3	384.6	408.5	23.9	~ 4
	MoO_3	5	383.9	408.9	25.0	# > 5

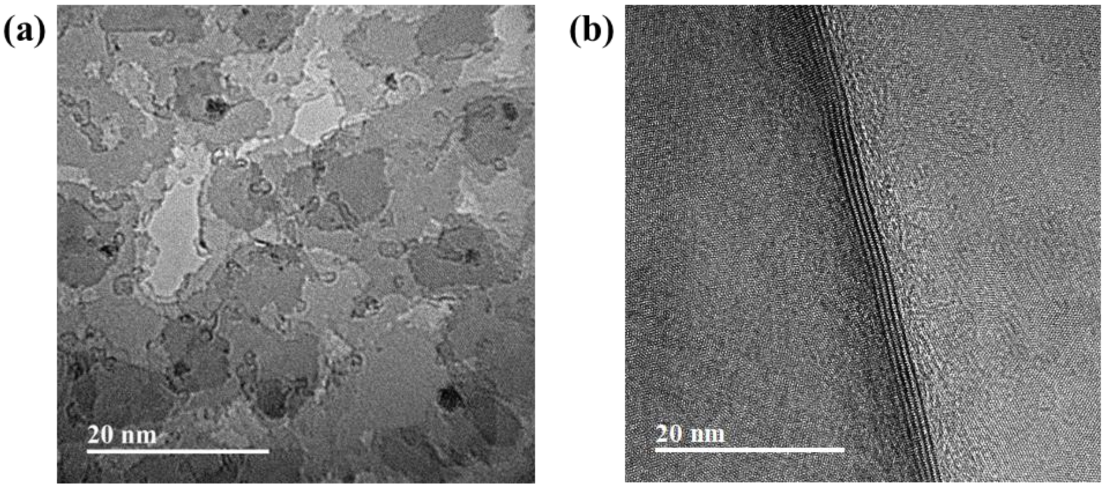

Figure S2. TEM images of synthesized WS_2 from **(a)** 1 nm and **(b)** 3 nm thick WO_3 thin films.

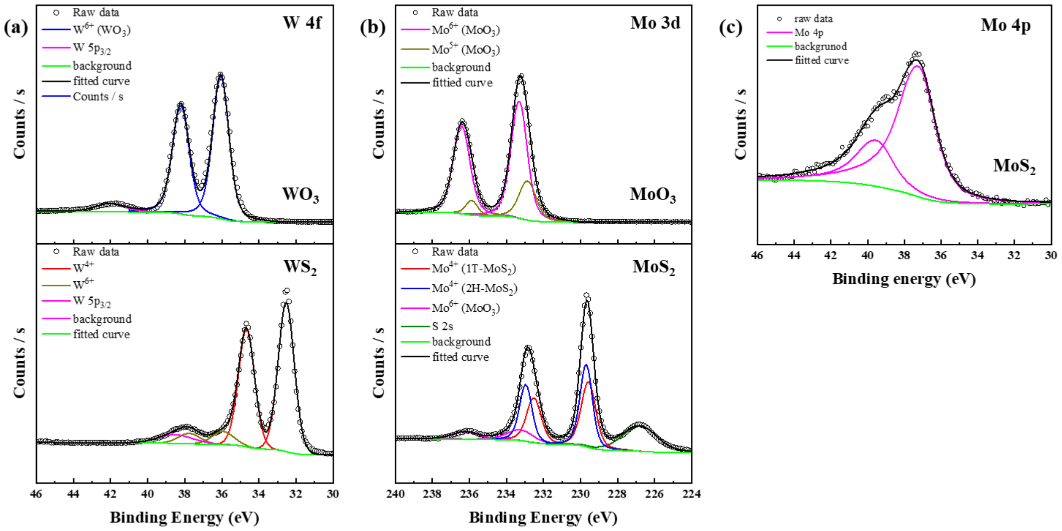

Figure S3. **(a)** Changes in W4f spectra before and after WS_2 synthesis, **(b)** Changes in Mo3d spectra before and after MoS_2 synthesis, **(c)** Mo4p spectra of MoS_2 films.

Table S2. Quantitative elemental analysis results and compositions from the XPS spectra.

Sample		W at.%	Mo at.%	S at.%	ratio S / (W+Mo)
Single	WS_2	32.8	-	67.2	2.0
Single	MoS_2	-	32.9	67.1	2.0
Hetero	Type I	14.8	25.4	59.7	1.5
Hetero	Type II	7.4	30.3	62.3	1.7
Hetero	Type III	9.5	46.3	44.2	0.8
